# Spatial patterns and links between microbial community composition and function in cyanobacterial mats

**DOI:** 10.3389/fmicb.2014.00406

**Published:** 2014-08-06

**Authors:** Mohammad A. A. Al-Najjar, Alban Ramette, Michael Kühl, Waleed Hamza, Judith M. Klatt, Lubos Polerecky

**Affiliations:** ^1^Microsensor Group, Max-Planck Institute for Marine MicrobiologyBremen, Germany; ^2^Marine Microbial Ecology Group, Red Sea Research Center, KAUSTThuwal, Saudi Arabia; ^3^Microbial Habitat Group, Max-Planck Institute for Marine MicrobiologyBremen, Germany; ^4^Marine Biological Section, Department of Biology, University of CopenhagenHelsingør, Denmark; ^5^Plant Functional Biology and Climate Change Cluster, University of Technology SydneySydney, NSW, Australia; ^6^Biology Department, UAE UniversityAl-Ain, UAE; ^7^Department of Earth Sciences – Geochemistry, Utrecht UniversityUtrecht, Netherlands

**Keywords:** spatial link between structure and function, photosynthetic microbial mats, imaging PAM, biogeography, hyperspectral imaging, microbial community structure

## Abstract

We imaged reflectance and variable fluorescence in 25 cyanobacterial mats from four distant sites around the globe to assess, at different scales of resolution, spatial variabilities in the physiological parameters characterizing their photosynthetic capacity, including the absorptivity by chlorophyll *a* (*A*_chl_), maximum quantum yield of photosynthesis (*Y*_max_), and light acclimation irradiance (*I*_k_). Generally, these parameters significantly varied within individual mats on a sub-millimeter scale, with about 2-fold higher variability in the vertical than in the horizontal direction. The average vertical profiles of *Y_max_* and *I*_k_ decreased with depth in the mat, while *A*_chl_ exhibited a sub-surface maximum. The within-mat variability was comparable to, but often larger than, the between-sites variability, whereas the within-site variabilities (i.e., between samples from the same site) were generally lowest. When compared based on averaged values of their photosynthetic parameters, mats clustered according to their site of origin. Similar clustering was found when the community composition of the mats' cyanobacterial layers were compared by automated ribosomal intergenic spacer analysis (ARISA), indicating a significant link between the microbial community composition and function. Although this link is likely the result of community adaptation to the prevailing site-specific environmental conditions, our present data is insufficient to identify the main factors determining these patterns. Nevertheless, this study demonstrates that the spatial variability in the photosynthetic capacity and light acclimation of benthic phototrophic microbial communities is at least as large on a sub-millimeter scale as it is on a global scale, and suggests that this pattern of variability scaling is similar for the microbial community composition.

## Introduction

A major challenge in microbial ecology is to understand how the structure, composition, and function of microbial communities are linked, how microbial communities are influenced by environmental conditions, and how they contribute to the local and global cycling of elements. Examples of microbial communities that have been extensively studied from all of these perspectives are cyanobacterial mats, which are highly compacted microbial ecosystems consisting of diverse phototrophic and heterotrophic populations (van Gemerden, 1993; Stal, 2000). The interest in their study stems from the generally accepted assumption that they represent a modern analog of the earliest complex ecosystems on Earth (Seckbach and Oren, 2010). Additionally, their vertically stratified and compact structure offers good possibilities for studying microbial and biogeochemical interactions in well-controlled laboratory mesocosms.

Environments that harbor cyanobacterial mats vary greatly with respect to parameters such as nutrient concentrations, temperature, salinity, or input of mineral particles. This variability leads to differences in the structure of the mats with respect to the density and distribution of microbial cells, content of inorganic particles, and characteristics of exopolymers that bind the mat matrix together. In spite of these differences, one feature that these environments have in common is their extremity with respect to at least one of the environmental parameters, typically salinity, temperature, or pH (Caumette et al., 1994; Garcia-Pichel et al., 1999; Des Marais, 2003; Abed et al., 2006; Seckbach and Oren, 2010). This environmental constraint is essential for excluding, or at least minimizing, the influence of grazing and bioturbation, which would otherwise disturb the perennial growth and laminated structure of the mats.

A typical feature of cyanobacterial mats are steep vertical gradients of physical and chemical parameters such as light, O_2_, pH, and H_2_S (van Gemerden, 1993; Stal, 2000). While the gradients in light intensity and spectral composition are the consequence of strong and wavelength-dependent absorption and scattering by photosynthetically active (photopigments) and inactive (mineral particles, organic detritus) components in the mat (Kühl and Jørgensen, 1992, 1994; Kühl et al., 1994), the steep chemical gradients form due to mass transfer limitation in a volume that is densely packed with active microbial cells (Kühl et al., 1996; Wieland and Kühl, 2000a; Jonkers et al., 2003; Garcia de Lomas et al., 2005).

A critical process in cyanobacterial mats is the photosynthetic activity of the cyanobacterial population, which occurs in the uppermost layer of the mat (so-called euphotic zone) and supports diverse heterotrophic populations in the mat ecosystem through the production of organic substrates and O_2_ (Nübel et al., 1999; Roeselers et al., 2007). Cyanobacterial photosynthesis depends on a number of environmental parameters, including temperature, salinity, concentration of nutrients, intensity and spectral quality of light, and exposure to H_2_S (Kühl, 1993, 2005; Kühl and Fenchel, 2000; Wieland et al., 2003; Pinckney et al., 2011). Since these parameters vary between different sites that harbor cyanobacterial mats as well as within the mats themselves (see above), it is expected that the rate and efficiency of photosynthesis in the mats will exhibit strong geographical as well as micrometer-scale variability. Although previous measurements demonstrated that photosynthetic rates and efficiency are strongly variable within mats (Kühl et al., 1994; Al-Najjar et al., 2010), presently it is not known how this within-mat variability compares to the variability between mats from different environments. Additionally, it is not known whether there is a link between the photosynthetic capacity in the euphotic zone of the mats and the composition of the corresponding microbial community, and to which extent are these properties of the mats determined by the parameters characterizing their environment.

To address these issues, we compared 25 samples of cyanobacterial mats collected from four distant geographical locations (United Arab Emirates, Australia, Brazil, and Spain) with respect to their photosynthetic capacity and microbial community composition. Our focus was on the cyanobacterial layer at the top of the mats, which was assumed to be a good proxy for the mats' photosynthetically active zone. We used absorptivity by chlorophyll *a* (*A*_chl_), maximum quantum yield of photosystem II (*Y*_max_), and light acclimation irradiance (*I*_k_) as parameters characterizing the photosynthetic capacity and adaptation of the cyanobacterial populations. These parameters were measured with a sub-millimeter spatial resolution across vertical sections of the cyanobacterial layers using hyper-spectral and variable chlorophyll fluorescence imaging. Differences in the microbial community composition of the cyanobacterial layers were quantified by automated ribosomal intergenic spacer analysis (ARISA). Possible links between the parameters characterizing the photosynthetic capacity, microbial community composition, and environmental parameters were identified using multivariate statistical methods. We hypothesized that the microbial community composition and photosynthetic capacity in the cyanobacterial layers are linked, and that the photosynthetic potential and light acclimation of the cyanobacterial populations are more strongly influenced by the steep vertical gradients within the mat ecosystem than by the environmental parameters characterizing their habitat.

## Materials and methods

### Samples

The studied cyanobacterial mats originated from four sites: an intertidal flat near Abu-Dhabi, UAE (AD mats), an intertidal flat in the Exmouth Gulf in Australia (AU mats; Lovelock et al., 2010; Adame et al., 2012), the hypersaline lake Lagoa Vermelha in Brazil (BR mats; Vasconcelos et al., 2006), and the hypersaline lake La Salada de Chiprana in Spain (SP mats; Jonkers et al., 2003). The mat samples were collected between 2003 and 2008 in at least two replicates from each site and incubated under artificial illumination (10 h light/14 h dark cycles, wavelength range 400–700 nm) and at approximately constant temperature until the measurements, which were conducted in 2009. More details about the collection sites and incubation conditions are given in Table 1. During the incubation, the appearance of the AD, AU, and BR mats did not change, whereas the SP mats gradually changed from thinly laminated structures (see Jonkers et al., 2003) to thicker structures featuring a loosen upper layer composed of a mixture of exopolymers and suspended particles.

**Table 1:** **Characteristics of the sampling sites, collection details, and incubation conditions for the studied microbial mats**.

**Site abbreviation**	**AD**	**AU**	**SP**	**BR**
**ENVIRONMENTAL PARAMETERS^a^**				
Type of site	Intertidal flat	Intertidal flat	Hypersaline lake	Lagoon (mixed with seawater)
Salinity range (‰)	35–200	35–50	78–90	40–150
Temperature (°C)	25–55	24–28	15–28	25–29
Inundation period	Few hours everyday	For ~6 h per month	Continuous	Continuous
Water depth when inundated (m)	1	2	1.5	1–2
Type of substrate	Very fine grained sediment	Very fine grained sediment	Very fine grained sediment	Organic material
N (mg/l)	2	840	1.2	NA^b^
P (mg/l)	0.03	96	0.04	0.23
**SAMPLE COLLECTION**				
Year	2007	2008	2003	2008
Geographic location	24°31′20″N,	22°43′7″S,	41°14′30″N,	22°53′15.2″S,
	54°26′50″E	114°34′6″E	0°10′50″W	42°6′38.14″W
Replicates	13	8	2	2
Within-site replicates separation (m)	~50	~10	~1^c^	~10
**INCUBATION CONDITIONS**				
Salinity range (‰)	35–200	35–50	80–90	35–200
T (°C):	26–28	26–28	20	26–28
Downwelling irradiance^d^ (μmol photons m^−2^ s^−1^):	380^e^	120–180^f^	240^g^	380^e^

a *Information extracted from literature: Al-Najjar et al. (2010) for AD mats; Lovelock et al. (2010) for AU mats; Jonkers et al. (2003) for SP mats; Vasconcelos et al. (2006) for BR mats*.

b *Value not measured*.

c *Taken from an aquarium where the mats were grown for ~6 years*.

d *Quantified by a calibrated PAR quantum irradiance sensor (LI-190 Quantum) connected to a light meter (LI-250, both from LI-COR Biosciences)*.

e *Light source: AQUALINE 10000, MH 400W, Germany*.

f *Light source: Envirolite, UK*.

g *Light source: cool white fluorescent tubes T8 (32W), Philips, Germany*.

### Measurement protocol

First, the mat samples were pre-incubated for about 12 h at room temperature and incident irradiance of 100 μmol photons m^−2^ s^−1^. Subsequently, they were vertically sectioned and immediately afterwards variable chlorophyll fluorescence and spectral reflectance were measured to characterize, respectively, the photosynthetic potential and pigments in the mats. This was done with a high spatial resolution (~20 μm) across the vertical sections of the mats using imaging cameras (see below). Immediately after imaging, cyanobacteria-dominated layers close to the mats surface were cut-off with a sterile scalpel and prepared for the ARISA (see below). Identification of these layers was based on their characteristic dark-green appearance, microscopic observations and the results of the imaging analyses, which were rapidly obtained by the image processing routines developed during this study (see below).

### PAM imaging of the variable chlorophyll fluorescence

Pulse amplitude modulated (PAM) imaging of the variable chlorophyll fluorescence was done with the Imaging-PAM system (Walz GmbH, Germany), using red light-emitting diodes for the excitation of the chlorophyll *a* fluorescence from cyanobacteria. For each mat, a vertical section of the mat sample was placed on its side in a Petri dish and covered with a few millimeters of seawater (salinity of 32, temperature 15°C). After 15 min of dark adaptation, images of the minimum (*F*_o_) and maximum (*F*_m_) fluorescence yields in the dark-adapted state were recorded. Subsequently, rapid light curves (RLC) (Schreiber et al., 1996, 1997) were measured by increasing the actinic irradiance from 0 to 1700 μmol photons m^−2^ s^−1^ in time intervals of 3 min for each irradiance and acquiring the fluorescence yield images under actinic illumination (*F*') and during the saturating pulse (*F*_m_′) at the end of each interval. The intensity and duration of the saturating pulse was ~2400 μmol photons m^−2^ s^−1^ and 0.8 s, respectively, and the irradiance levels of the actinic light were calibrated using a PAR (400–700 nm) quantum irradiance sensor (LI-190 Quantum) connected to a light meter (LI-250, both from LI-COR Biosciences) positioned in the same place as the mat sample. As the sample was relatively small and it was lying on its side, irradiance was evenly distributed across the vertical section of the mat.

### Imaging of chlorophyll a absorptivity

In addition to variable fluorescence imaging, the Imaging-PAM system was used to image reflectance of the mats in the red (*R*_r_) and near-infrared (*R*_nir_) region. These images were used to calculate the chlorophyll *a* absorptivity as *A*_chl_ = (*R*_nir_ − *R*_r_)/*R*_nir_, which was taken as a proxy for chlorophyll *a* concentration in the mats. Because the spectral resolution of these measurements was insufficient, chlorophyll *a* absorptivity was additionally quantified by hyper-spectral imaging (Kühl and Polerecky, 2008; Polerecky et al., 2009). Specifically, each mat sample in the Petri dish was placed on a motorized stage, illuminated with a halogen bulb (Philips, type 6423) emitting in the visible to near-infrared range (400–900 nm), and scanned with a hyper-spectral imaging system (VNIR-100, Themis, Themis Vision, USA). Spectral normalization was achieved by scanning a gray reference standard with 40% reflectance (SRS-40-020, Labsphere Inc., USA). After verification that the reflectance spectrum had a characteristic chl *a* edge in the wavelength range of 700–720 nm and a flat plateau above 720 nm (see, e.g., Polerecky et al., 2009), the image of chl *a* absorptivity was calculated as *A*_chl_ = (*R*_750_ − *R*_675_)/*R*_750_, where *R*_750_ and *R*_675_ are the reflectance images measured at 750 nm (no absorption by chl *a*) and 675 nm (maximal chl *a* absorption), respectively. No difference was found between the chl *a* absorptivity values obtained by hyper-spectral imaging and by the Imaging-PAM system (data not shown). Therefore, the latter were used in the subsequent analysis, as they allowed perfect alignment with the variable fluorescence images.

### DNA extraction and ARISA

DNA from the cyanobacterial layers in the studied mats was extracted and purified using the UltraClean soil DNA isolation kit (MO BIO Laboratories, Inc., Carlsbad, CA, USA) according to the manufacturer's instructions. For each mat sample, PCR (50 μl) were conducted in triplicates and contained 1× PCR buffer (Promega, Madison, WI, USA), 2.5 mM MgCl_2_ (Promega), 0.25 mM of 40 mM dNTP mix (Promega), bovine serum albumin (3 μg/μl, final concentration), 25 ng extracted DNA, 400 nM each of universal primer ITSF (5′-GTCGTAACAAGGTAGCCGTA-3′) and eubacterial ITSReub (5′-GCCAAGGCATCCACC-3′; Cardinale et al., 2004) labeled with the phosphoramidite dye HEX, and 0.05 units GoTaq polymerase (Promega). All subsequent steps, including the PCR protocol, purity of the PCR products, labeling of the products, discrimination of the PCR-amplified fragments via capillary electrophoresis (ABI PRISM 3130*xl* Genetic Analyzer, Applied Biosystems) and the subsequent statistical analysis of ARISA profiles (quality control, binning, merging) were done as previously described (Boer et al., 2009; Ramette, 2009).

### Processing and analysis of the fluorescence yield images

**Figure 1:**
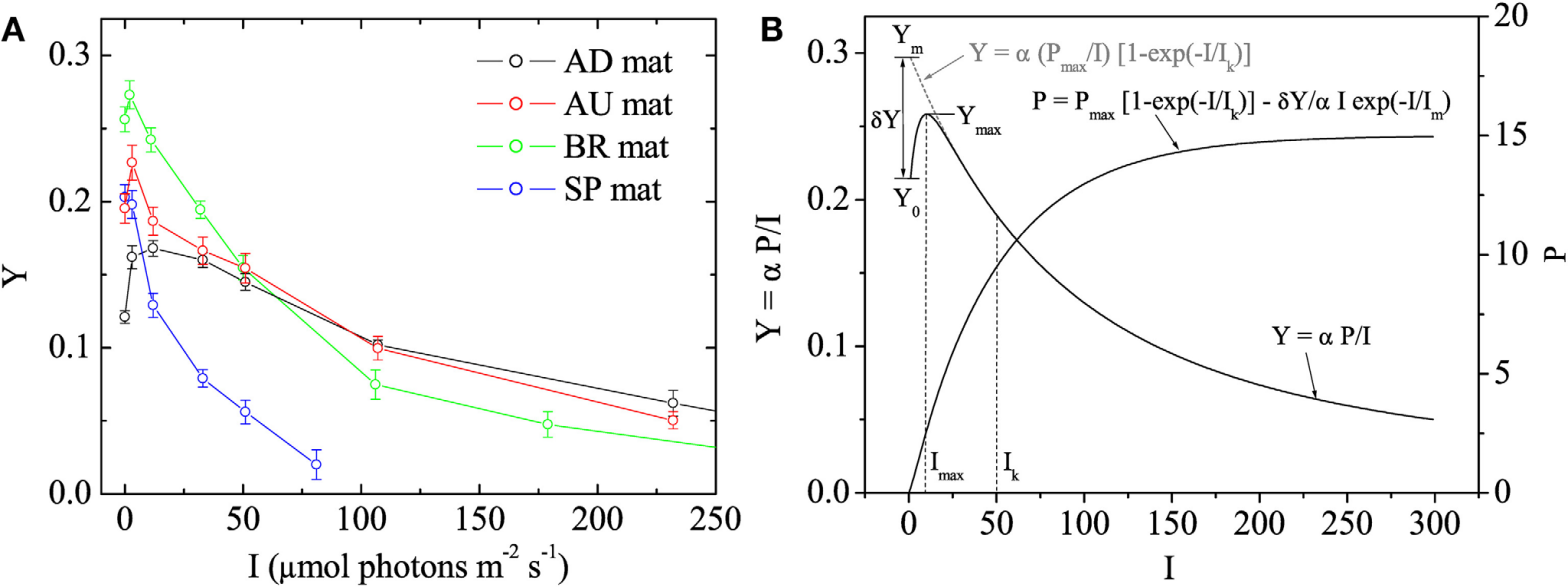
**(A)** Examples of quantum yields of photosystem II, *Y*, in the studied cyanobacterial mats, as measured by pulse amplitude modulated (PAM) imaging at different irradiances, *I*. Symbols and error bars represent, respectively, the mean and SD calculated from 5 × 5 pixels in the image. **(B)** Mathematical model describing the *Y* vs. *I* relationship. The main features of the relationship are annotated. The corresponding relationship between photosynthesis, *P*, and *I* is also shown.

Using the fluorescence yield images, the quantum yield of PSII in the dark-adapted state was calculated as *Y*_0_ = (*F*_m_ − *F*_o_)/*F*_m_ and the effective quantum yield of PSII at a given actinic irradiance, *I* > 0, as *Y* = (*F*_m_′ − *F*')/*F*_m_′. Subsequently, the values of *Y* were plotted as a function of *I* to determine the maximum effective quantum yield of PSII (denoted as *Y*_max_) and the actinic irradiance at which *Y* reached *Y*_max_ (denoted as *I*_max_). Based on the theoretical background of the saturation pulse method (Baker, 2008), *Y*_max_ represents the maximal photosynthetic potential of the cyanobacterial population in the mat. In plants, *Y* decreases monotonously with *I* and this maximal potential is reached in the dark-adapted state, i.e., *Y*_max_ = *Y*_0_ and *I*_max_ = 0 (White and Critchley, 1999). However, in our measurements with cyanobacterial mats the *Y-I* relationship was not monotonous and these parameters were typically related as *Y*_max_ > *Y*_0_ and *I*_max_ > 0 (Figure 1), which is why we additionally determined also *I*_max_.

In photosynthesis research, light acclimation is determined from the relationship between the rate of photosynthesis, *P*, and irradiance, *I*. This so-called *P-I* curve is close to linear at low irradiance levels and approaches saturation at high irradiance levels. The actual value of the light acclimation irradiance (denoted here as *I*_k_) depends on the mathematical model that describes it, and is typically obtained by fitting the *P-I* data (see, e.g., Platt and Jassby, 1976). To obtain *I*_k_ from our data, we assumed that the parameter *Y* measured by the saturation pulse method is given by *Y* = α *P*/*I*, where α is a proportionality constant whose value is not important in this study (but see Campbell et al., 1998), and considered the following model to describe the *P-I* relationship:

(1) $$P(I)=P_{\text{max}}[1-\exp\text{​}(\text{​​}-I/I_{\text{k}})]-\delta Y/\alpha I\exp\text{​}(\text{​​}-I/I_{\text{m}}).$$

The first term in this model is equivalent to that proposed by Webb et al. (1974), whereas the second term was necessary to describe the non-monotonous relationship between *Y* and *I* observed in this study (Figure 1). Using Equation (1) and assuming that *I*_m_ ≪ *I*_k_, which was the case in our measurements, *Y* can be written approximately as

(2) $$Y\approx Y_{\text{m}}(I_{\text{k}}/I)[1-\exp\text{​}(\text{​​}-I/I_{\text{k}})]-\delta Y\exp\text{​}(\text{​​}-I/I_{\text{max}}),$$

where *Y*_m_ = *Y*_max_/(1 − *I*_max_/*I*_k_), δ*Y* = *Y*_m_ − *Y*_0_ and *I*_max_ ≈ *I*_m_ if *I*_m_ ≪ *I*_k_ (see Figure 1B). Thus, by determining the values of *Y*_0_, *Y*_max_, and *I*_max_ and fitting the rest of the measured *Y-I* values with the model in Equation (2), it was possible to determine the remaining fitting parameter *I*_k_. Data processing required for this analysis, including quantification of the images of *Y*_max_, *I*_max_, and *I*_k_, their variabilities across the vertical and horizontal directions as well as average vertical and horizontal profiles, was done in Matlab (The MathWorks Inc., Natick, MA) using the program Look@PAM developed during this study. This program is available on the internet (http://www.microsen-wiki.net/pamimaging:lookatpam).

### Statistical analyses

The significance of univariate response data as a function of categorical factors was tested using one-way analysis of variance (ANOVA), after verifying the normality (Shapiro–Wilk normality test) of the response variable at *p* = 0.05. Community differences were visualized by non-metric multidimensional scaling (MDS) ordination based on the Bray–Curtis dissimilarity matrix between samples, and significance of community differences between groups of samples was determined by Analysis of Similarity (ANOSIM) tests. Prior to analyzing ARISA profiles conjointly with functional or environmental variables, a consensus community profile was obtained for each sample by merging the triplicate ARISA PCR and by considering an OTU present if it appeared at least twice among the triplicates (Ramette, 2009). The merged table was Hellinger-transformed to minimize the effects of the strongly right-skewed distribution curve (Ramette, 2007). To assess the link between the microbial community composition and function, the Procrustes superimposition approach was used to estimate the concordance of scores originating from two independent ordinations after rotating, translating, and dilating one of them, while keeping the other ordination coordinates constant (Gower, 1975). Significance of the rotation statistic was assessed by Monte-Carlo permutations (Peres-Neto and Jackson, 2001). All statistical tests were carried out with the statistical platform *R* (http://cran.r-project.org/) and multivariate community analyses with the *vegan* package (Oksanen et al., 2012).

## Results

**Figure 2:**
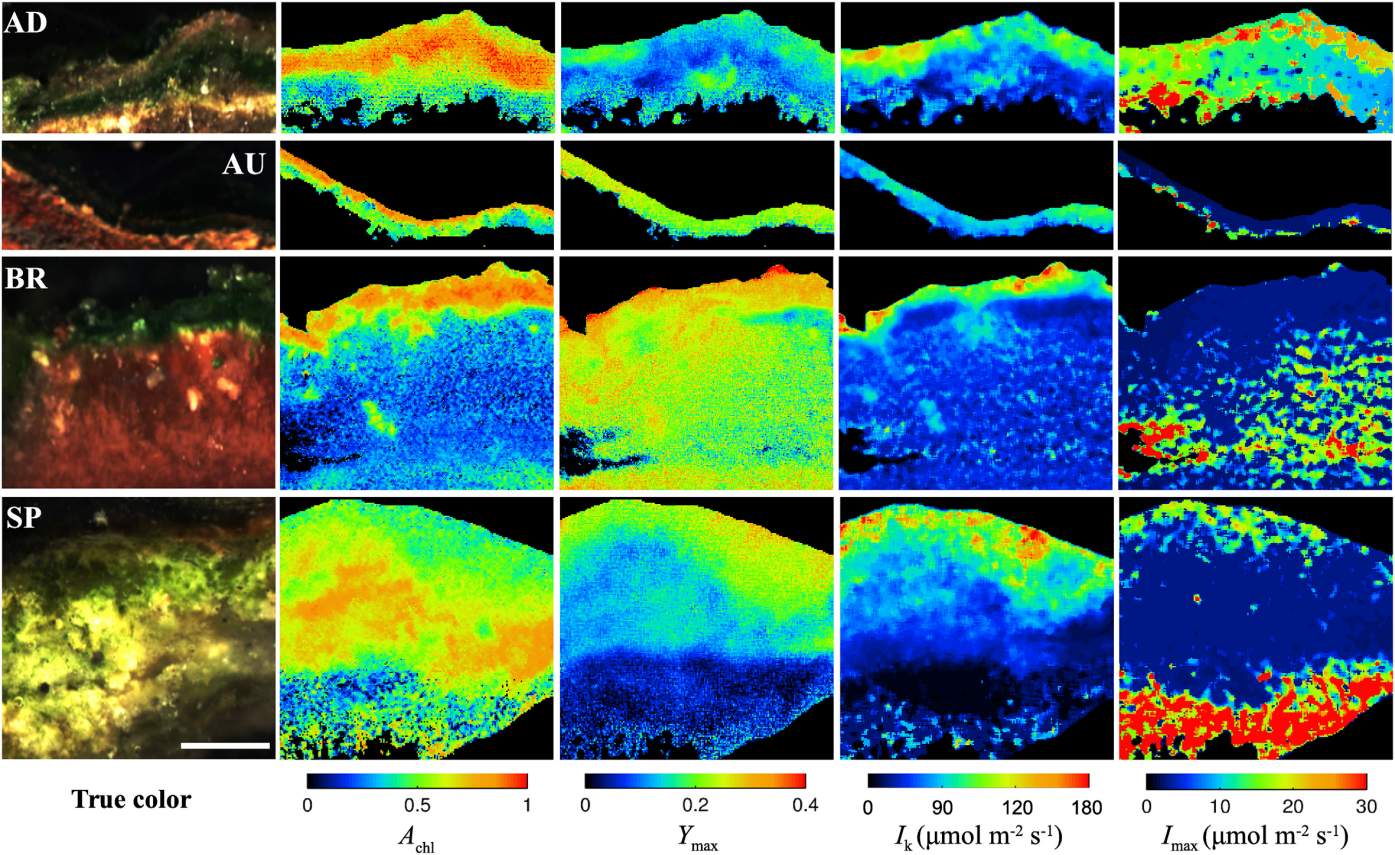
**Example images of the AD, AU, BR, and SP mats obtained by hyperspectral imaging (column “true color”), reflectance imaging (*A*_chl_), and PAM imaging (*Y*_max_, *I*_k_, *I*_max_). Scale bar is 1 mm**.

All studied mats had a clear laminated structure, with a characteristic dark-green layer at or close to the mat surface in each of them (see examples of true color images in Figure 2). The AD and AU mats had a brown layer and a red layer underneath the dark-green layer, while the deepest layer was black. Additionally, the AD mats were covered by a thin orange gelatinous layer. The BR mats showed a different layering pattern with an upper dark-green layer followed by a thicker dark-pink gelatinous layer. The SP mats had a similar structure as the AD mats, except the distinct layers were thicker and the surface gelatinous layer had whitish to light-brown appearance.

Hyperspectral imaging revealed that the dark-green layer in the mats had a pronounced absorption at wavelengths corresponding to the maximal absorption by chlorophyll *a* (675 nm) and phycocyanin (625 nm; Figure S1). Since these pigments are characteristic for cyanobacteria, we could be confident that the dark-green layer contained abundant cyanobacterial populations and could therefore be referred to as the cyanobacterial layer. This conclusion was supported by microscopic observations (data not shown).

### Variability of physiological parameters on different spatial scales

Imaging of *A*_chl_, *Y*_max_, *I*_max_, and *I*_k_ in mats collected from different sites made it possible to investigate how the variability in these physiological parameters changes depending on the scale at which it is determined, including the micrometer-scale (within-mat variability), meter-scale (within-site variability), and global-scale (between-site variability). The within-mat variability was quantified in three ways: as standard deviation (SD) of the values from the entire image of the cyanobacterial layer and as SD of the average vertical and horizontal profiles. The within-site variability was calculated as SD of the average values over the cyanobacterial layer in each mat from the respective site, while the global variability as SD of the average values for each site.

**Figure 3:**
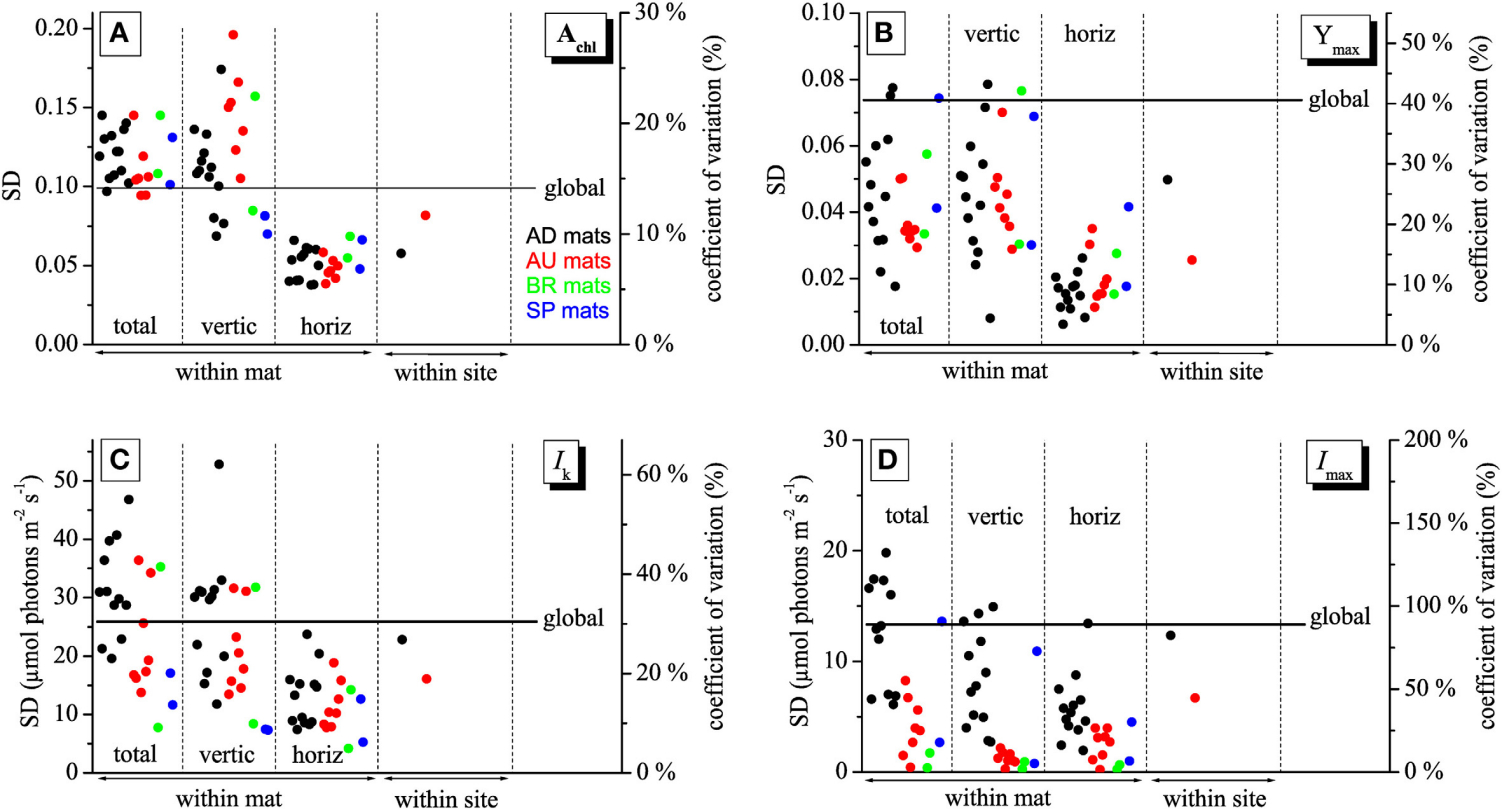
**Variabilities of the physiological parameters *A*_chl_**(A)**, *Y*_max_**(B)**, *I*_k_**(C)**, and *I*_max_**(D)** calculated at different spatial scales, including the micrometer (within-mat), meter (within-site), and global (between sites) scales**. See text for details. The variabilities are expressed as standard deviations, SD (left axis), and as a coefficient of variation (right axis).

As demonstrated by the images, all studied physiological parameters exhibited pronounced micrometer-scale variability within the cyanobacterial layer of the studied mats (Figure 2). Two-factorial analysis of variance performed on individual images of *A*_chl_, *Y*_max_, and *I*_k_, using vertical and horizontal position in the mat as factors, revealed that the percentage of the total within-mat variance explained by the vertical position (25–30% for *A*_chl_, 45–50% for *Y*_max_, and 30–35% for *I*_k_) was about 1.5 to 2-fold larger than the percentage of variance explained by the horizontal position (15–20% for *A*_chl_, 15–30% for *Y*_max_, 15–25% for *I*_k_). This was consistent with the comparison of the SD for the average vertical and horizontal profiles, which showed that the former were about 2-fold larger than the latter (Figures 3A–C). Thus, on average, the vertical variability in *A*_chl_, *Y*_max_, and *I*_k_ was about twice as high as the horizontal one. A significant portion of the within-mat variance was explained by the interaction between the vertical and horizontal position (55–60% for *A*_chl_, 30–35% for *Y*_max_, and 45–50% for *I*_k_), consistent with the clearly visible variation of the vertical profiles of these parameters along the horizontal direction for each individual mat sample (Figure 2). With respect to *I*_max_, the vertical and horizontal variabilities were similar (Figure 3D), each explaining about 10% of the total within-mat variability, while the remaining 80% was explained by their interaction. The within-mat variability of *I*_max_ in the AD mats was significantly larger than in the other mats (Figure 3D), which was mainly because the *I*_max_ values were generally larger in the AD mats.

Average vertical profiles of *A*_chl_ for AD, BR, and SP mats were characterized by a subsurface maximum, whereas a monotonous decrease with depth was observed for AU mats (Figure S2A). Average *Y*_max_ and *I*_k_ generally decreased with depth (Figures S2B,C) and were significantly correlated (*p* < 0.001) for each mat sample. Spatial trends in *I*_max_ were not so clear, having patchy distributions for some mats while on average increasing with depth for others (Figure 2 and Figure S2D). Depending on the mats, vertical variation across the cyanobacterial layer, expressed as a coefficient of variation (SD/mean) of the average vertical profile, reached 10–30% for *A*_chl_, 5–45% for *Y*_max_, 10–60% for *I*_k_, and 5–100% for *I*_max_ (Figure 3).

The within-site variability of *A*_chl_ calculated for the mats from the AD and AU sites was lower than the within-mat variabilities calculated for the individual mats from these sites (Figure 3A). This was often the case also for *Y*_max_, *I*_k_, and *I*_max_, but sometimes the within-mat variability was lower than the within-site one (Figures 3B–D). Such comparison could not be done reliably for the BR and SP mats because of the limited number of replicates (*N* = 2) for these two sites.

The global-scale variability was larger than the within-site variability for all measured physiological parameters (Figure 3). In contrast, in many cases it was lower than the within-mat variability. This relationship was most pronounced for *A*_chl_ and *I*_k_, where it was observed in 21 (for *A*_chl_) and 12 (for *I*_k_) out of 24 mat samples (Figures 3A,C). Clearly, this was primarily due to the pronounced variability in the vertical direction, whereas the horizontal within-mat variability was almost always lower than the global-scale variability. On the other hand, the global-scale variability in *Y*_max_ and *I*_max_ was mostly larger than the within-mat variability, except for a few AD mats (Figures 3B,D). The global-scale variability, expressed as a coefficient of variation, was about 15% for *A*_chl_, 40% for *Y*_max_, 30% for *I*_k_, and 80% for *I*_max_ (Figure 3).

### Clustering of mats based on average physiological parameters

**Figure 4:**
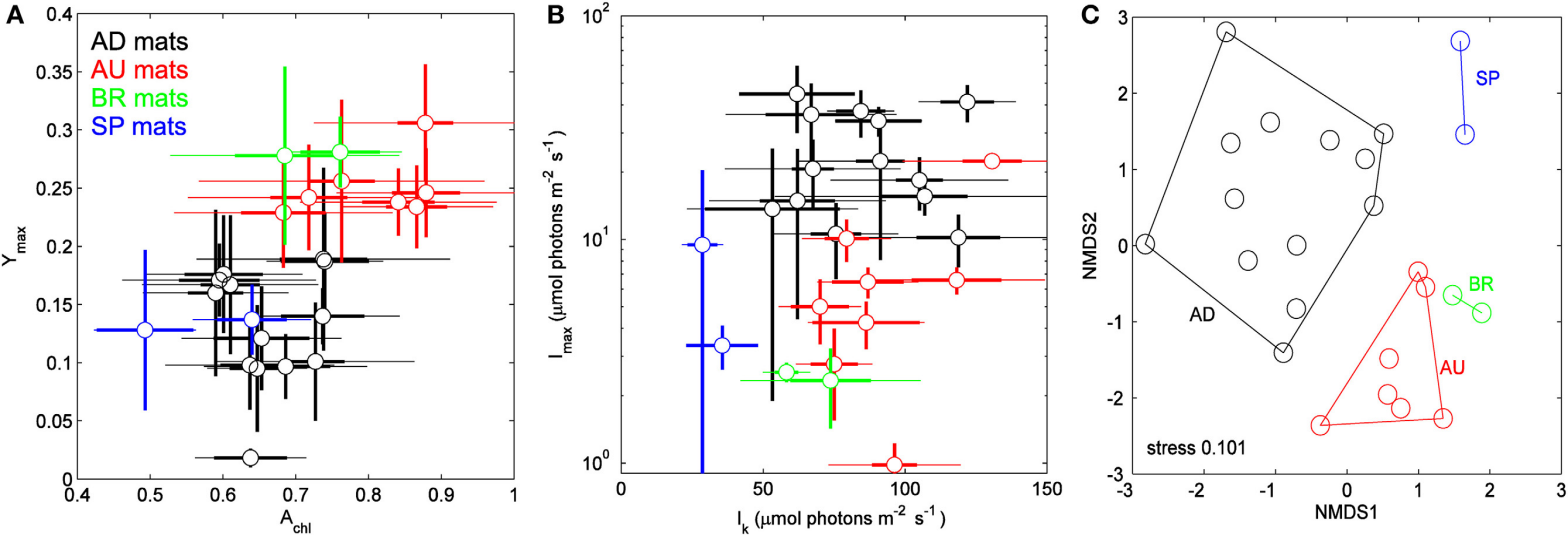
**(A,B)** Relationships between physiological parameters *A*_chl_, *Y*_max_, *I*_k_, and *I*_max_ in the studied cyanobacterial mats. Symbols represent averages over the cyanobacterial layers, thin and thick error-bars depict the total and vertical variability (expressed as standard deviation, SD) within the layers, respectively. **(C)** Multidimensional scaling plot of the distance matrix calculated based on the average values of the parameters shown in **(A,B)** using Euclidean metric.

Physiological parameters *A*_chl_, *Y*_max_, *I*_max_, and *I*_k_, when averaged over the cyanobacterial layers, varied significantly between the sampled sites (Figure 4, Table 2). For example, AU and BR mats had, on average, about 2-fold larger *Y*_max_ than AD and SP mats, SP mats had the lowest *I*_k_, whereas AD mats had the largest values of *I*_max_. When a distance matrix was calculated from the average values of *A*_chl_, *Y*_max_, *I*_k_, and *I*_max_ using Euclidean metric, its visualization in an MDS plot revealed clear clustering of the mats according to the site of their origin (Figure 4C).

**Table 2:** **Average values and standard deviations of the physiological parameters characterizing the mats from the different sites**.

	**AD**	**AU**	**BR**	**SP**	***p*^a^**
*A*_chl_	0.66±0.06 [2]^b^	0.80±0.08 [1]	0.72±0.05 [1–2]	0.57±0.10 [2]	0.0004
*Y*_max_	0.13±0.05 [2]	0.25±0.03 [1]	0.280±0.002 [1]	0.13±0.01 [2]	8 × 10^−6^
*I*_k_(μmol m^−2^ s^−1^)	85±23 [1]	88±16 [1]	66±11 [1–2]	32±5 [2]	0.01
*I*_max_ (μmol m^−2^ s^−1^)	24±12 [1]	5±3 [2]	2.4±0.2 [2]	6±4 [2]	1 × 10^−5^

a *Probability that the means between the different sites are equal, as determined by ANOVA. The I_max_ values were log-transformed before ANOVA to ensure variance homogeneity*.

b *Means ranked as [1] are significantly larger than means ranked as [2], means ranked as [1–2] are not significantly different from those ranked as [1] and [2]*.

### Clustering of mats based on the microbial community composition

**Figure 5:**
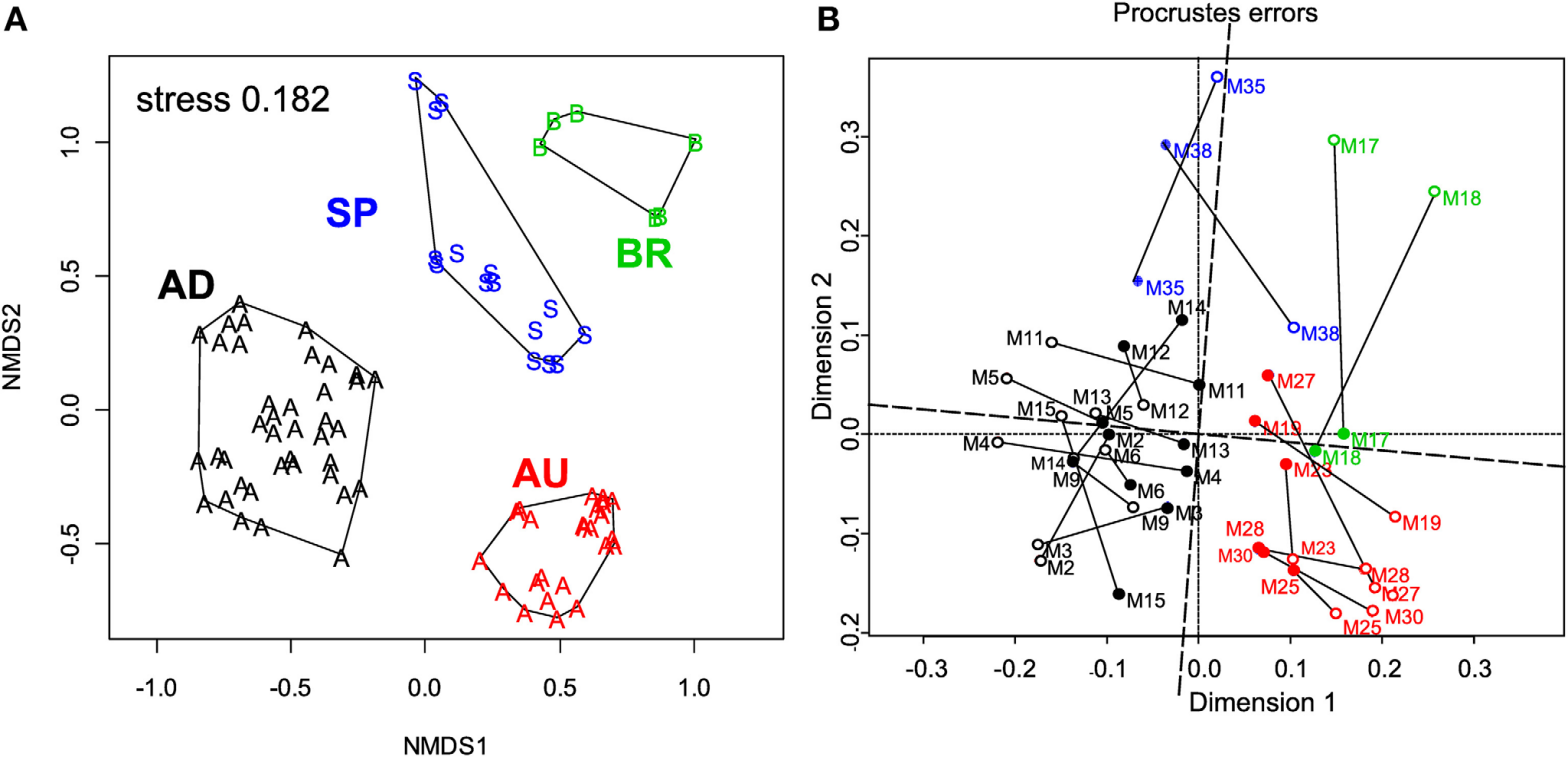
**(A)** Variation in the microbial community composition within the cyanobacterial layers of the studied mats. A Bray–Curtis dissimilarity matrix was calculated based on ARISA community profiles and is displayed in a 2D ordination space (associated stress value of 0.182). Grouping lines were added for each site *a posteriori* to highlight the site specificity in the community patterns. **(B)** Procrustes analysis of the link between the composition and function of the microbial communities in the studied mats. Open symbols represent the ARISA-based NMDS data, while the closed ones represent the functional data (Figure 4). Note the small rotation angle between the two sets of ordination axes. AD mats (m2–m15); BR mats (m17–m18); AU mats (m19–m30); SP mats (m35 and m38).

ARISA fingerprinting revealed that the microbial communities in the mats from a given site were more similar to each other than to those from other sites (Figure 5A). This marked endemism was further supported by significant ANOSIM test (*P* = 0.0001). Overall, the sampled bacterial communities shared between 18 and 34% OTUs, with 84 out of 398 OTUs (21.1%) found everywhere (i.e., at least in one mat from a given site) and 314 OTUs being mat-specific.

### Link between community composition and function

To assess the degree of concordance between community composition and their potential function (photosynthetic capacity), we compared the bacterial community composition as depicted by the NMDS plot in Figure 5A with that of the configuration of *A*_chl_, *Y*_max_, *I*_k_, and *I*_max_ (Figure 4C) by Procrustes analyses. A significant concordance between ordinations was found (*r* = 0.605, *P* = 0.002, based on 1000 permutations; Figure 5B), suggesting that distinct communities were associated with distinct (potential) functions.

### Environmental parameters

Most parameters characterizing the habitat of the studied mats were obtained from literature and are summarized in Table 1. The sites strikingly differed with respect to nutrient concentrations in the overlying water, which were extremely high for the AU mats. This was very likely because the sediments at this site contained large biomass of insect larvae (alive and dead), which could be a source of nutrients (Behie et al., 2012). Additionally, the site was inundated in brief and infrequent intervals (during a spring tide). Another notable difference between the sites was due to the salinity fluctuations, which were very large at the AD and BR sites.

## Discussion

### Scaling of variability in physiological parameters

The imaging approach used in this study enabled us to assess how the spatial variability in parameters characterizing the photosynthetic potential in cyanobacterial mats scales depending on the sampling resolution (from sub-millimeter to thousands of km). Our main result is that this scaling pattern differed depending on the studied parameter, with chlorophyll *a* absorptivity (*A*_chl_) and acclimation intensity (*I*_k_) having on average the largest variability on the micrometer-scale (mostly in the vertical direction) while the maximum quantum yield of PSII (*Y*_max_) and the irradiance at which this maximum yield is reached (*I*_max_) were most variable on the global scale (between sites). This contrast suggests that the different physiological parameters are controlled by different environmental factors, as discussed below.

The pronounced micrometer-scale vertical variability in *A*_chl_ was most likely due to the combined effects of light and nutrients. The growth of photosynthetic microbial mats is typically limited by nutrients, with most nutrients assimilated in the cyanobacterial layer originating from organic matter remineralization underneath the layer (Jonkers et al., 2003). On the other hand, light quantity strongly attenuates with depth due to intense absorption by photopigments and abiotic components of the mat matrix (Kühl and Jørgensen, 1992, 1994; Kühl et al., 1994). Thus, because of the opposing gradients in light and nutrients, cyanobacterial growth at the top and bottom of the cyanobacterial layer is likely limited by nutrients and light, respectively, whereas a location with an optimal supply of light and nutrients is somewhere in the middle. This suggests that the cyanobacterial biomass should have a maximum somewhere around this optimal location. Assuming that the measured chlorophyll *a* absorptivity, *A*_chl_, is a proxy for cyanobacterial biomass, our results are consistent with this interpretation: while *A*_chl_ had a clear subsurface maximum in mats from sites where the N and P concentrations in the overlying water were low (AD, SP, BR), it decreased sharply from the surface in the mats from the AU site, where the nutrient concentrations in the overlying water were very high (Table 1, Figure S2). This latter characteristic of the AU site was most likely also the main factor responsible for on average the highest values of *A*_chl_ (Figure 4A, Table 2) and its vertical variability (Figure 3A) in the AU mats as compared to the mats from the other sites. Additional explanation for the observed within-mat variability in *A*_chl_ is the tendency of phototrophic cells to have a larger cellular pigment content when grown at lower irradiances (Falkowski, 1980; Kirk, 2011). Thus, at least in the mats from AD, SP, and BR, the observed subsurface maximum in *A*_chl_ could additionally be due to this adaptation of cyanobacterial cells to light limitation that progressively increases with depth in the mat. Together, this suggests that the average cyanobacterial biomass in the cyanobacterial layer of the studied mats was mostly determined by the overlying water nutrient content, whereas the micrometer-scale distribution of the biomass, and possibly also of the average pigment content in the cyanobacterial cells, was additionally shaped by light.

With respect to the light acclimation intensity, *I*_k_, light availability was likely the most important factor that determined both its micrometer-scale and global-scale variation. Assuming that cyanobacteria adapt to local light conditions, the sharp decrease in light intensity with depth in mats should result in cyanobacterial populations in deeper parts of the euphotic zone being acclimated to lower light intensities. This is what we generally observed for mats from all sites (Figure S2). Although we do not have reliable data on site-specific downwelling irradiances, the observation that the global-scale variability of *I*_k_ was comparable to, and in many occasions lower than, the micrometer-scale variability suggested that light variation within the photosynthetically active cyanobacterial layer was similar or larger than the variation in the average light dose received by the mats from the different sites.

Although the micrometer-scale variability of the maximum effective quantum yield, *Y*_max_, was strongly positively correlated with the light acclimation intensity for each studied mat (see e.g., Figure S2), light availability is not a likely factor that controls its spatial distribution. This is primarily because *Y*_max_ is, by definition, a proxy for the maximum photosynthetic *potential*, which is reached at light intensities that are considerably lower than the acclimation intensity (in the dark for plants, at *I*_max_ ≪ *I*_k_ for the cyanobacterial populations in this study; Figure 1). Since the quantum yield of PSII determined by the PAM measurement of variable chlorophyll fluorescence relates to the redox state of the plastoquinone (PQ) pool in the photosynthetic electron transport chain (Allen, 2003), parameters that affect this redox state are likely affecting also *Y*_max_. In the context of cyanobacterial mats, H_2_S and O_2_ are likely candidates.

The response of cyanobacterial photosynthesis to H_2_S is well-documented (Cohen et al., 1986; Jørgensen et al., 1986; Miller and Bebout, 2004), with several species being able to perform anoxygenic photosynthesis using H_2_S as the electron donor. In such cyanobacteria, H_2_S oxidation is facilitated by the activity of sulfide-quinone-reductase (SQR) (Bronstein et al., 2000; Griesbeck et al., 2000), which reduces the PQ molecule using the electrons transferred from H_2_S and can therefore decrease the apparent quantum yield of PSII. Our preliminary experiments with an axenic cyanobacterial culture embedded in agarose showed that exposure to H_2_S in the μM range lead to a rapid decrease in the quantum yield *Y*, both in the dark and light, and the yield recovery in the light occurred only after H_2_S decreased below a threshold in the μM range (Figure S3). A similar negative effect of H_2_S on *Y* was observed in other cyanobacterial systems (e.g., living stromatolites; Kromkamp et al., 2007). Therefore, the pronounced decrease in *Y*_max_ with depth in the cyanobacterial layer may be due to the steep increase in H_2_S in this layer at low-light conditions, which is a well-documented phenomenon in mats (e.g., Wieland and Kühl, 2000b; Jonkers et al., 2003; Garcia de Lomas et al., 2005; Al-Thani et al., 2014).

The effect of O_2_ on *Y* is likely because photosynthetic and respiratory electron transport chains in a cyanobacterial cell share three electron carriers in the thylakoid membrane (PQ, cytochrome b_6_f and the terminal oxidases; Vermaas, 2001), which makes O_2_ an important electron acceptor for electrons from the reduced PQ (chlororespiration) and also for those coming from PSI via ferredoxin (Mehler reaction) (Schreiber et al., 2002). Presence of O_2_ can therefore contribute to a partial oxidation of the PQ pool, which could manifest itself as an increase in the apparent *Y*. The steep decrease in O_2_ with depth, which is typical for cyanobacterial mats under low light conditions (Wieland and Kühl, 2000a, 2006; Al-Najjar et al., 2012), could therefore additionally be responsible for the observed decline of *Y*_max_ with depth.

In addition to environmental parameters mentioned above, part of the observed spatial variability of *Y*_max_, *I*_k_ and *A*_chl_ could be due to artifacts linked to autofluorescence (for *Y*_max_ and *I*_k_) and absorption (for *A*_chl_) by photosynthetically inactive components in the cyanobacterial layer, such as dead/inactive cyanobacterial cells, pigment degradation products or mineral particles. Such components are abundant in the cyanobacterial layer of microbial mats (e.g., Kühl et al., 1994; Al-Najjar et al., 2012), and because their fluorescence is not variable but possibly comparable to that from the PSII of photosynthetically active cells, the detected quantum yield of PSII can appear lower. Similarly, their absorption in the same wavelength range as chlorophyll *a* could increase the apparent *A*_chl_. These effects may have contributed to the differences in the “baseline” of these parameters and thus also their apparent global variability. The importance of this contribution could, however, not be estimated based on our present data.

In contrast to the monotonous decrease in the effective quantum yield, *Y*, with the actinic irradiance, *I*, which is characteristic for plants (White and Critchley, 1999) and eukaryotic algae (Flameling and Kromkamp, 1998), our measurements in cyanobacterial mats showed that the relationship between *Y* and *I* was not monotonous (Figure 1). Instead, *Y* reached a maximum at actinic intensities *I*_max_ that varied in the range from one to several tens of μmol photons m^−2^ s^−1^ (Figure 4B). The spatial patterns for *I*_max_ within a given mat were not very clear (see, e.g., Figure 2), primarily because of the noise in the fluorescence yield images. Although we were able to find a suitable mathematical expression for this relationship (Equation 2), further research is required to understand why *Y* reached a maximum at intensities *I*_max_ > 0 and what controls this behavior.

### Implications for microbial ecology

Our imaging data demonstrate that the variability of a specific function (here photosynthesis) in a microbial community (here the cyanobacterial layer) can be at least as large on the micrometer-scale within the community as it is on the global scale between communities from different locations. As discussed above, this is primarily due to the strong within-mat variability of the physico-chemical parameters that control the function. Furthermore, our comparison of the cyanobacterial layers based on their average functional (photosynthetic) potential and microbial community composition revealed that the mats clustered according to their site of origin (Figures 4C, 5A) and that the functional and compositional data were significantly linked (Figure 5B). Based on these results we expect that if physico-chemical parameters exhibit pronounced variability within a microbial community, which is common in transport-limited systems such microbial mats and sediments, the community composition will also vary at least as much on the micrometer-scale (within community) as it does on the global scale (between communities). This implies that analyses of the microbial community composition that ignore this micrometer-scale variability (e.g., by sampling bulk volumes of sediments rather than layer-by-layer) may have a limited value in identifying correlations between the microbial composition, function and the corresponding environmental settings. In other words, when sampling microbial communities in order to identify these correlations, the prefix “micro” should refer not only to the size of the inhabitants but also to the required spatial resolution of sampling.

Interestingly, despite the large micrometer-scale variability of the functional potential within each cyanobacterial layer community, distinct communities from a given site were on average characterized by a distinct, albeit highly variable, functional potential (Figure 4). This suggests that out of the environmental parameters discussed in the previous section, there are possibly one or more factors that have the most dominant influence on the composition and function of the studied cyanobacterial mats. Unfortunately, these dominant factors could not be identified from the limited dataset presented in this study.

### Conflict of interest statement

The authors declare that the research was conducted in the absence of any commercial or financial relationships that could be construed as a potential conflict of interest.
