# Supplementary material for: Spatial patterns and links between microbial community composition and function in cyanobacterial mats
Source: Front Microbiol. 2014 Aug 6;5:406. doi: 10.3389/fmicb.2014.00406 (PMC4123731; doi:10.3389/fmicb.2014.00406)
Supplement: Supplementary file 1 [file Presentation1.PDF]

Spatial patterns and links between microbial community composition and function in cyanobacterial mats

Mohammad A. A. Al-Najjar, Alban Ramette, Michael Kühl, Waleed Hamza,
Judith M. Klatt, Lubos Polerecky

Supplementary Information

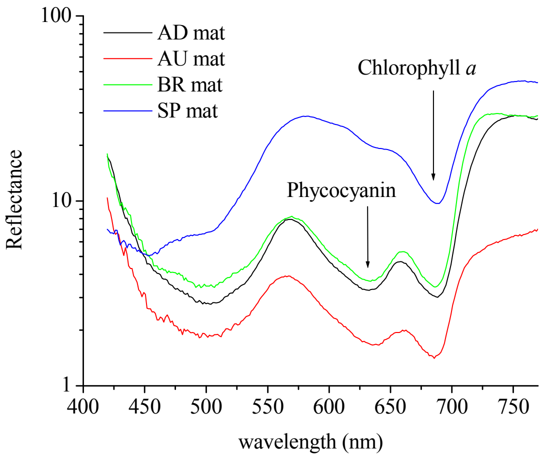

Figure S1: Hyperspectral imaging of the studied cyanobacterial mats. Shown are representative examples of reflectance spectra in selected points from the cyanobacterial layers in mats from each site. Arrows indicate wavelengths of maximal absorption by chlorophyll *a* and phycocyanin.

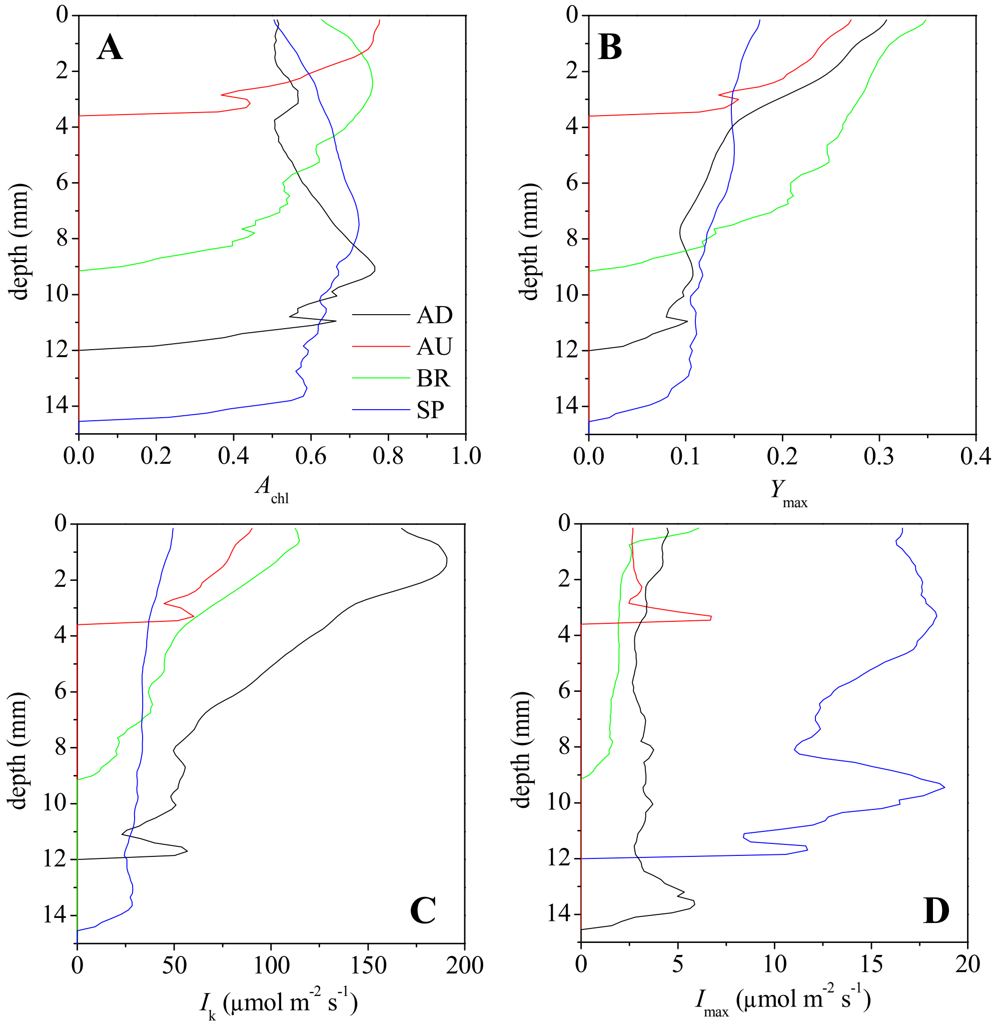

Figure S2: Examples of average vertical profiles of physiological parameters in the studied cyanobacterial mats. The profiles were calculated from the images shown in Figure 2. The depth where the values reach zero corresponds to the thickness of the cyanobacterial layer.

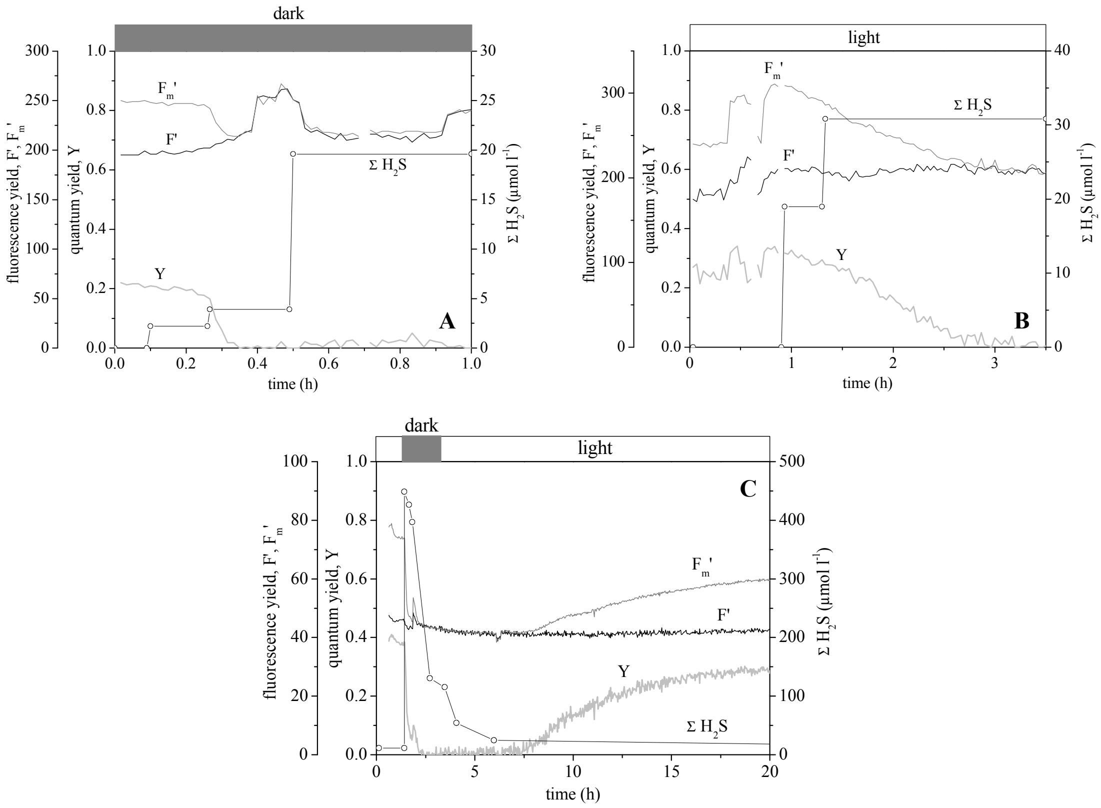

Figure S3: Effect of hydrogen sulfide (H_2S) on the variable fluorescence yield and quantum yield of PSII in an artificial cyanobacterial biofilm. The studied cyanobacteria (*Microcoleus* sp.; culture provided by Prof. U. Fischer, University of Bremen, Germany) were grown in the ASNIII medium (Ripka et al. 1979) with salinity of 32. During the exponential growth phase the *Microcoleus* sp. filaments were concentrated by gentle centrifugation, mixed with a low melting-point (~37 ºC) agarose (Sigma-Aldrich, Germany) and cast into a 1 mm thick mold prepared from two glass slides. The filaments-agarose mixture was let solidify by cooling at room temperature for 2-3 min, which resulted in 1 mm thick artificial biofilms that could be easily manipulated. Oxygen microsensor measurements in the light and in the dark confirmed that the biofilms were photosynthetically active. Subsequently, variable fluorescence (at actinic illumination, F', and during the saturating pulse, F_m') from the biofilms was measured using the Diving-PAM instrument (Heinz Walz GmbH, Germany). This was done with the biofilms placed in a closed chamber into which the Diving-PAM fiber optic was inserted through a rubber stopper. The overlying medium was made anoxic (i.e., O_2 concentrations <1 µM, as determined by an O_2 microelectrode) by purging with N_2 prior to the measurement. In the first experiment,

H_2S was added to the medium in steps until a decrease in the quantum yield of PSII, *Y*, was observed. In the dark, *Y* decreased within minutes towards 0 when the H_2S concentration in the medium exceeded about 4 µM (panel A). In the light (downwelling irradiance 150 µmol photons m^{-2} s^{-1}), *Y* started to decrease at H_2S concentrations above 20-30 µM and the rate of decrease was about 10-fold slower than in the dark (panel B). In the second experiment the concentration of H_2S added to the medium was initially high and was subsequently decreased by adding H_2S-free medium. Recovery of *Y* over several hours occurred only in the light and when the H_2S concentrations in the medium dropped below about 10 µM (panel C).

Supplementary references

Rippka, R., Deruelles, J., Waterbury, J.B., Herdman, M., and Stanier, R.Y. (1979). Generic assignments, strain histories and properties of pure cultures of cyanobacteria. *Journal of General Microbiology* 111**,** 1-61.
